# COVID-19-Associated Mortality in US Veterans with and without SARS-CoV-2 Infection

**DOI:** 10.3390/ijerph18168486

**Published:** 2021-08-11

**Authors:** Ayako Suzuki, Jimmy T. Efird, Thomas S. Redding, Andrew D. Thompson, Ashlyn M. Press, Christina D. Williams, Christopher J. Hostler, Christine M. Hunt

**Affiliations:** 1 Division of Gastroenterology, Duke University, Durham, NC 27710, USA; Christine.Hunt@va.gov; 2 Division of Gastroenterology, Durham VA Medical Center, Durham, NC 27705, USA; 3 VA Cooperative Studies Program Epidemiology Center, Durham VA Health Care System, Durham, NC 27705, USA; Jimmy.Efird@va.gov (J.T.E.); Thomas.Redding28@va.gov (T.S.R.IV); Andrew.Thompson3@va.gov (A.D.T.J.); ashlyn.press@va.gov (A.M.P.); Christina.Williams4@va.gov (C.D.W.); 4 Department of Medicine, Duke University, Durham, NC 27710, USA; 5 Duke Cancer Institute, Duke University School of Medicine, Duke University Health Care System, Durham, NC 27710, USA; 6 Division of Infectious Diseases, Duke University School of Medicine, Durham, NC 27710, USA; Christopher.Hostler@va.gov; 7 Infectious Diseases Section, Durham VA Health Care System, Durham, NC 27705, USA

**Keywords:** COVID-19, risk factor, mortality, smoking

## Abstract

Background: We performed an observational Veterans Health Administration cohort analysis to assess how risk factors affect 30-day mortality in SARS-CoV-2-infected subjects relative to those uninfected. While the risk factors for coronavirus disease 2019 (COVID-19) have been extensively studied, these have been seldom compared with uninfected referents. Methods: We analyzed 341,166 White/Black male veterans tested for SARS-CoV-2 from March 1 to September 10, 2020. The relative risk of 30-day mortality was computed for age, race, ethnicity, BMI, smoking status, and alcohol use disorder in infected and uninfected subjects separately. The difference in relative risk was then evaluated between infected and uninfected subjects. All the analyses were performed considering clinical confounders. Results: In this cohort, 7% were SARS-CoV-2-positive. Age >60 and overweight/obesity were associated with a dose-related increased mortality risk among infected patients relative to those uninfected. In contrast, relative to never smoking, current smoking was associated with a decreased mortality among infected and an increased mortality in uninfected, yielding a reduced mortality risk among infected relative to uninfected. Alcohol use disorder was also associated with decreased mortality risk in infected relative to the uninfected. Conclusions: Age, BMI, smoking, and alcohol use disorder affect 30-day mortality in SARS-CoV-2-infected subjects differently from uninfected referents. Advanced age and overweight/obesity were associated with increased mortality risk among infected men, while current smoking and alcohol use disorder were associated with lower mortality risk among infected men, when compared with those uninfected.

## 1. Introduction

Since emerging in December 2019, the severe acute respiratory syndrome coronavirus 2 (SARS-CoV-2) has rapidly evolved into the global coronavirus disease-19 (COVID-19) pandemic, requiring prompt risk management [1,2]. COVID-19 is now a leading cause of death in the US with its high infectivity, transmission from asymptomatic or presymptomatic infected patients, and incomplete containment and mitigation strategies [3,4]. Earlier epidemiological studies demonstrated that COVID-19 mortality increases among those of advanced age, male sex, Black race, and Latinx ethnicity [5,6,7]. Many of those with fatal infection had obesity, hypertension, diabetes, heart disease, chronic liver disease, or kidney disease [8,9,10,11]. With limited patient numbers, most case series have evaluated risk factors independently and have not compared outcomes in uninfected patients tested for SARS-CoV-2 as the referent (control) group. Providing care to nine million veterans, the Veterans Health Administration (VHA) is the largest integrated US healthcare system; its electronic health record database is well designed to identify and characterize COVID-19 risk modifiers [5,10,12]. 

In this observational cohort analysis, we assessed the contribution of demographics, current smoking, and alcohol use disorder to 30-day mortality following the diagnosis of SARS-CoV-2 infection. More specifically, among those tested for SARS-CoV-2 during the study period, we compared the relative risk of 30-day mortality for age, race, ethnicity, body mass index (BMI), smoking status, and alcohol use disorder in SARS-CoV-2-positive patients with that in SARS-CoV-2-negative referents. We then examined the effect of multifactorial interactions on mortality to assess whether the effects of the risk factors on mortality were modified by other risk factors. Our goal was to evaluate the mortality risk of demographic characteristics, current smoking, and alcohol use disorder in SARS-CoV-2-infected cohort relative to the uninfected, to better understand how these factors interact with SARS-CoV-2 infection and influence 30-day mortality. Our findings will inform future preventive strategies and early intervention for high-risk groups to reduce severe outcomes in future pandemics.

## 2. Materials and Methods

### 2.1. Study Design and Data Source

We developed a well-characterized patient cohort tested for SARS-CoV-2 using the regularly updated VA COVID-19 Shared Data Resource, the VA Corporate Data Warehouse (CDW), and the VHA Informatics and Computing Infrastructure (VINCI). The COVID-19 Shared Data Resource includes information on all patients tested for SARS-CoV-2 within the VA or who tested positive outside VA with information of the positive test recorded in VA clinical notes [13]. At the time of SARS-CoV-2 testing, patients were classified as SARS-CoV-2-positive or -negative referents (i.e., infected or uninfected, respectively) and followed to assess 30-day mortality.

The specific aims were to (1) assess the relative contribution of demographics, current smoking, and alcohol use disorder to 30-day mortality in SARS-CoV-2-positive subjects versus negative referents; (2) evaluate multifactorial interactions in the association with 30-day mortality by stratified analyses. The Durham Veterans Affairs (VA) Health Care System Institutional Review Board approved this study protocol.

### 2.2. Cohort Definition

We defined SARS-CoV-2-positive and -negative veterans using all available SARS-CoV-2 nucleic acid amplification tests (NAAT) and antigen tests from March 1 to September 10, 2020. SARS-CoV-2 antibody tests were excluded from this analysis; our cohort was classified by the presence or absence of SARS-CoV-2 infection. SARS-CoV-2 antibody tests were excluded from this analysis; our cohort was classified by the presence or absence of SARS-CoV-2 infection. The SARS-CoV-2-infected cohort was composed of all positive subjects during the study period, using the collection date of the first positive result as the index date. Repeated positive results from the same individuals were excluded from this analysis. The SARS-CoV-2-uninfected cohort was composed of subjects whose tests were all negative during the study period. The index date for the SARS-CoV-2-negative referents was the last negative collection date during the study period.

During the study period, only subjects who were symptomatic or were required to have a SARS-CoV-2 test for hospitalization or certain procedures (e.g., risk of respiratory exposure) underwent testing per VA COVID-19 guideline. The majority of SARS-CoV-2-positive subjects were symptomatic (i.e., COVID-19). 

Based on our preliminary cohort evaluation, we excluded female veterans from the analysis owing to their low mortality rates and as they comprised less than 10% of the population, which would preclude our planned stratified risk factor analyses to evaluate risk disparities by sex. For these same reasons, racial minorities (other than Black) were excluded. 

### 2.3. Study Variables Created for the COVID-19 Cohort

Outcome variables: The primary outcome was 30-day overall mortality after the index date. We also evaluated infection rate, post-index hospitalization (hospitalized after index date: yes or no), length of stay (days of the first hospitalization only), and mechanical ventilator use (yes or no) for descriptive analysis only. The infection rate was defined as the number of SARS-CoV-2-positive subjects per overall subject tests for SARS-CoV-2. This analysis did not account for hospitalizations, mechanical ventilator use, or deaths that occurred outside the VHA or beyond 30 days post-index date. 

Predictor variables: We analyzed six predictor variables: age, race, ethnicity, BMI, smoking status, and alcohol use disorder. Demographic information was obtained from the CDW. When sex and race/ethnicity information from different time points was inconsistent within individuals, this inconsistency was reconciled using existing algorithms [14]. Efficient maximum likelihood estimates for incomplete data (demographics and smoking) were obtained using the expectation–maximization (EM) algorithm [15]. For BMI and smoking status, data recorded preceding and closest to the index date were retrieved from the CDW. Alcohol use disorder was defined using International Classification of Diseases (ICD)-10 codes; subjects who had any ICD-10 codes indicative of alcohol use disorder within two years before the index date, either from inpatient or outpatient records, were considered to have alcohol use disorder. 

Age (years) and BMI (kg/m^2^) were categorized, instead of treating them as continuous variables, to detect a possible nonlinear association. Race and ethnicity were categorical: White vs. Black and Latinx vs. non-Latinx. Smoking status was recorded as current, former, or never and was classified into current smoking vs. others for the analysis. Alcohol use disorder was treated as binary (yes/no). 

Covariates: We considered locations of VA facilities and calendar month of diagnosis (i.e., index date) as covariates to adjust for geographic and temporal variations in infection rates, resources, and patient care. Geographic locations were classified into three categories (East-Coast, Mid-West, and Pacific-Mountain), and calendar month of index date was treated as a categorical variable. To adjust for baseline mortality risk, we computed the Charlson Comorbidity Index score using relevant ICD-10 codes within one year before index date and treated it as a covariate. Age categories were also adjusted for in the models. 

Other variables: Variables (yes/no) of baseline comorbidities (e.g., chronic disease conditions relevant to the VHA patient population) were created based on applicable ICD-10 codes within two years before the index date, including codes from inpatient and outpatient records. They were only used to describe the study cohort in this analysis. 

### 2.4. Statistical Analysis 

The contribution of age, race, ethnicity, BMI, smoking status, and alcohol use disorder to 30-day mortality was assessed in the SARS-CoV-2-positive patients and the SARS-CoV-2-negative referents, considering age, geographic location, calendar month of diagnosis, and baseline Charlson Comorbidity Index as covariates. Multifactorial interactions were then assessed using stratified analysis. 

Categorical variables were expressed as frequency and percentage; continuous variables were reported as median and interquartile range (IQR). Chi-square and Deuchler–Wilcoxon tests were used to compare the distribution of clinical characteristics between dead and recovered patients, by SARS-CoV-2-positive and -negative status. 

Adjusted relative risk (aRR) and 95% confidence intervals (CIs) for 30-day mortality were computed in SARS-CoV-2-infected and -uninfected groups for age groups, race (White vs. Black), ethnicity, BMI categories, smoking status, and alcohol use disorder (yes vs. no) using log-linear methods [16,17,18]. Covariates for the analysis were age (≤60, 61–70, 71–80, >80), region (Pacific-Mountain, Mid-West, East-Coast), month of index date (March, April, May, June, July, August/September), and Charlson Comorbidity Index (0, 1–2, 3–4, 5+). An interaction between a variable and SARS-CoV-2 status was then statistically tested to assess its contribution to mortality in the SARS-CoV-2-infected group, relative to mortality in the SARS-CoV-2-uninfected group. The risk difference between SARS-CoV-2-infected and -uninfected groups was computed as the difference in adjusted RR, using a logarithmic scale. *P* for linear trend of RR estimates was estimated using the Lagrangian multiplier test [19]. 

We then performed a stratified analysis to evaluate multifactorial interactions after dichotomizing variables: age (≤70 vs. >70); Black vs. White; BMI (<25 vs. ≥25); current smoking (yes vs. no); alcohol use disorder (yes vs. no). Limited Latinx patient numbers resulted in insufficient statistical power to include the Latinx ethnicity in the stratified analysis. Adjusted RR and the difference in adjusted RR between SARS-CoV-2-infected and -uninfected groups were computed in each stratum. Interactions between a variable and COVID-19 status as well as between strata were then statistically tested [20]. We used the same set of covariates for the stratified analysis. 

Adjustment for multiplicity was performed by the Hochberg–Bonferroni procedure [21]. Study results were rounded using the Goldilocks (Efron–Whittemore) method [22]. *p*-values < 0.05 were considered as statistically significant. 

## 3. Results

### 3.1. Cohort Characteristics

This national study cohort included 341,166 men, in whom 22,777 (7%) tested SARS-CoV-2-positive. The overall cohort was predominantly White (76%) and over age 60 (214,083 (63%)); 35,570 [10%] were of Latinx ethnicity. The overall prevalence of obesity (BMI ≥30), current smokers, and alcohol use disorder was 33%, 21%, and 18%, respectively. Prevalent comorbidities in the overall cohort included hypertension (63%), hyperlipidemia (59%), mental illness (51%), atherosclerosis (35%), and type II diabetes mellitus (34%). The prevalence of 30-day mortality in the overall cohort was 3%. 

Clinical characteristics by SARS-CoV-2 infection status are summarized in Supplemental Table S1. The key differentiating characteristics were a lower median age, a higher prevalence of Black race and Latinx ethnicity, a higher median BMI, and a lower prevalence of comorbidities, alcohol use disorder, and current smokers in SARS-CoV-2-infected versus -uninfected patients (Supplemental Table S1). Of note, among the 71,570 (21%) overall current smokers, 2617 (11%) were infected and 68,953 (22%) were uninfected. Current smokers had lower rates of infection (4%) compared with former (7%), or never smokers (9%) (Supplemental Table S1). 

In SARS-CoV-2-infected and -uninfected groups, 7% and 3% exhibited 30-day mortality, respectively (Table 1). In infected and uninfected patients, 30-day mortality was associated with older age, a lower prevalence of Latinx ethnicity, a lower median BMI, and a lower prevalence of current smokers (Table 1). In addition, 30-day mortality was associated with a lower prevalence of alcohol use disorder among infected patients, while a lower prevalence of Black race was observed in those who were uninfected (Table 1). 

### 3.2. Risk Factors Affecting Mortality in SARS-CoV-2 Infected Relative to Uninfected

**Figure 1:**
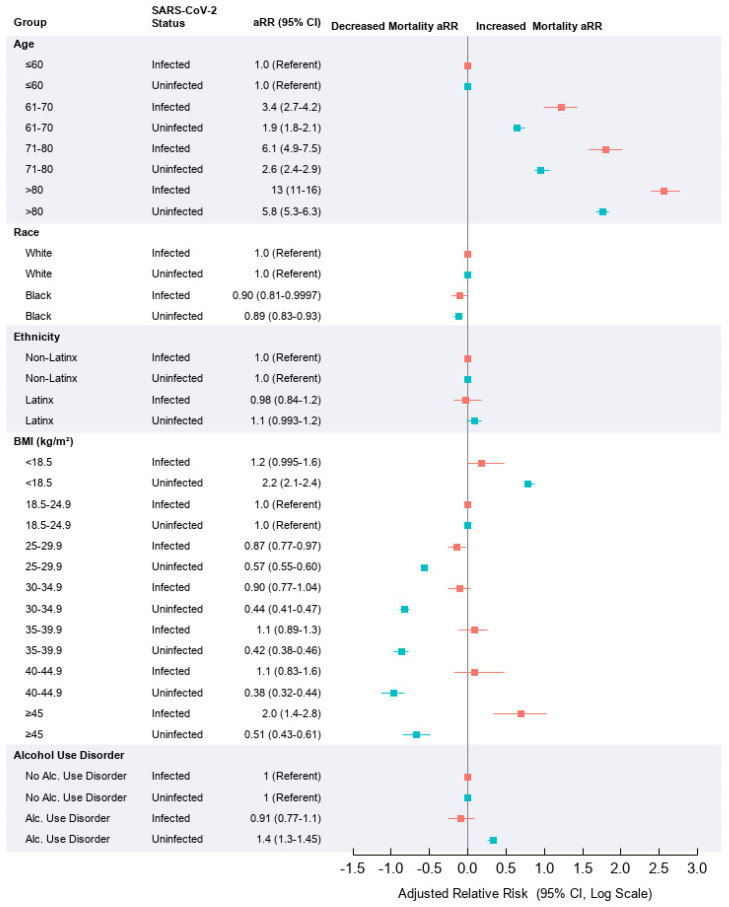

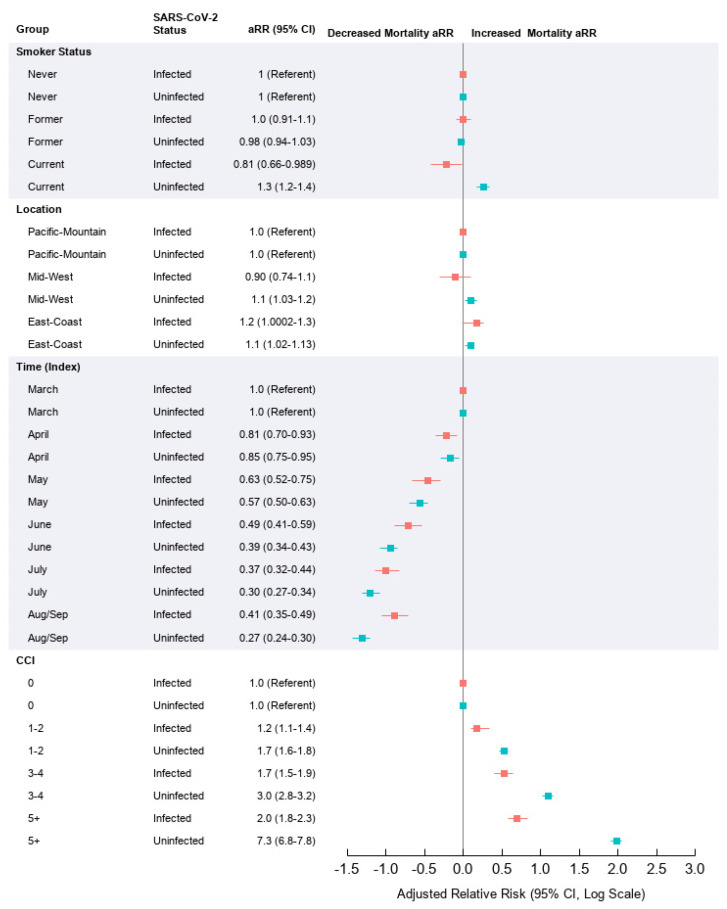
Forest plot of selected characteristics and adjusted relative risk of 30-day mortality by SARS-CoV-2 infection status among male veterans (n = 341,166). Adjusted relative risk of 30-day mortality in infected (red) and uninfected (blue) cohorts by study variable. Models were adjusted for age, location, time, and Charlson Comorbidity Index (unless the indicated characteristic). aRR = adjusted relative risk (Le Cam–Stein shrinkage). BMI = body mass index (kg/m^2^). CCI = Charlson Comorbidity Index.

The adjusted relative risk (aRR) of 30-day mortality for the study variables in the infected and uninfected groups and the aRR comparisons between the groups are summarized in Figure 1 and Supplemental Table S2. Relative to age ≤ 60 years, age over 60 significantly increased mortality in a dose-related manner among those infected and uninfected, with aRR of mortality highest in those over age 80 (aRR, 13; 95% CI, 11–16 in infected and aRR, 5.8; 95% CI, 5.3–6.3 in uninfected). When compared between groups, age over 60 years increased aRR in those infected to a greater extent than in those uninfected (*P* for interaction (*P*Int) < 0.0001) (Figure 1; Supplemental Table S2). Morbid obesity with BMI ≥ 45 showed an increased mortality relative to normal BMI (18.5–24.9) only among those infected (aRR, 2.0; 95% CI, 1.4–2.8). When compared between groups, overweight/obesity (BMI ≥25) progressively increased aRR of mortality among infected relative to the uninfected (*P*Int <0.0001), with a 290% increased aRR with BMI ≥ 45.

Relative to never smoking, current smoking was associated with a decreased mortality in those infected (aRR, 0.81; 95% CI, 0.66–0.989) and an increased mortality in uninfected patients (aRR, 1.3; 95% CI, 1.2–1.4), yielding a 56% decrease in aRR among those infected vs. uninfected (*P*Int <0.0001). Alcohol use disorder showed a tendency toward an association with decreased mortality among infected (aRR, 0.91; 95% CI, 0.77–1.1) and increased mortality in those uninfected (aRR, 1.4; 95% CI, 1.3–1.45), exhibiting a 57% reduction of aRR among infected vs. uninfected (*P*Int < 0.0001). 

Compared with White race, Black race was associated with a modestly reduced mortality and no interaction with SARS-CoV-2 status. Ethnicity was not significantly associated with mortality. Overall, the stratified analyses showed consistent effects of old age, overweight/obesity, current smoking, and alcohol use disorder (Supplemental Table S3). Further adjustment for all the variables included in Supplemental Table S2 in a pairwise fashion did not substantively alter the effects of the study variables (data not shown). 

## 4. Discussion

In this national cohort of over 340,000 men tested for SARS-CoV-2, advanced age and overweight/obesity disproportionally increased the relative risk of mortality among infected vs. uninfected men. Interestingly, we found a lower rate of SARS-CoV-2 infection in current smokers compared with never smokers or former smokers. Relative to never smokers, infected current smokers consistently exhibited an association with lower mortality across the strata while uninfected current smokers displayed an association with increased mortality (Supplemental Table S3). Men with alcohol use disorder also exhibited a lower relative risk of mortality in infected vs. uninfected patients. In contrast, race and ethnicity did not disproportionally affect mortality in infected vs. uninfected patients. Ethnicity did not affect mortality. Compared with White race, Black race was associated with a similar lower mortality in both infected and uninfected patients. East Coast residence conferred a significantly increased mortality risk among infected patients, as did the timing of infection early in the pandemic, coinciding with a high prevalence of infection. The highest prevalence of infection in March was associated with the highest mortality, which declined over time in both infected and uninfected patients. This high mortality in those uninfected was associated with notably higher Charlson Comorbidity Index score, compared with the infected group.

Our findings support and extend those of other large COVID-19 studies in US and UK integrated health systems [5,6,11,23], with a heightened mortality risk seen with advanced age, overweight/obesity, and at times of high disease prevalence. Specifically, the notable decrease in mortality from March to September 2020 observed in our VHA study in both infected and uninfected patients mirrored that of another large national study of hospitalized SARS-CoV-2-positive and -negative patients at multiple medical centers during this timeframe [24]. This suggests that the pandemic surge overwhelmed critical care resources, contributing to an overall increased mortality. 

In the U.S., smoking is estimated to cause approximately 500,000 deaths annually [25]. Smokers have an overall increased risk of respiratory infections and severe outcomes [26]. Smokers suffer a three-fold higher adjusted risk of hospitalization for community-acquired pneumonia than that of nonsmokers, independent of comorbidity [27]. Therefore, one would expect an increased risk of SARS-CoV-2 infections and mortality among smokers. Some COVID-19 studies have reported smokers to exhibit a higher risk of mortality; however, these studies tend to be smaller and many did not adjust for age and comorbidities [28,29]. Prior VHA studies examining only SARS-CoV-2-positive patients (without SARS-CoV-2-negative referents) report no association of smoking with higher 30-day mortality or adverse outcomes in adjusted analyses [5,30]. When compared with SARS-CoV-2-negative referents, infected current smokers in our study exhibited a clinically significant lower risk of SARS-CoV-2 infection and mortality. The overall prevalence of current smokers in this study cohort was 21%, which is higher than the reported prevalence of current smokers among veterans, 15.5% [31]. Our higher prevalence of current smoking may reflect men in our analysis who more recently accessed the VA healthcare system. Furthermore, most of the population tested for SARS-CoV-2 had symptoms or were sick enough to be hospitalized or receive an invasive procedure/intervention during the pandemic. Smokers are associated with higher comorbidities (e.g., COPD and atherosclerosis), which could also explain the higher prevalence of current smokers in the cohort. Nonetheless, our smoking findings should be interpreted with caution, considering potential selection bias. 

Our analysis is consistent with multiple large COVID-19 studies in which smoking decreased the risk of hospitalization [8], ICU admission [8,32], and case fatality [11], as well as a meta-analysis [33] reporting that current smokers were at reduced risk of SARS-CoV-2 infection. Our lower risk of mortality among current smokers compared with never smokers (aRR, 0.81; 95% CI, 0.66–0.989) parallels that of the UK OpenSAFELY study (aHR: 0.89 (0.82–0.97)), similarly drawn from a population of millions [11]. These findings are further supported by a well-characterized study of a SARS-CoV-2 outbreak on a French Naval nuclear aircraft carrier affecting most of nearly 1700 young crewmembers, in whom nearly half (48%) smoked [34]. A dose-related protective effect of smoking was observed, with the lowest rate of SARS-CoV-2 infection seen in those smoking the highest number of cigarettes daily, in comparison with those who did not smoke daily [34], which further supports a potential causal association between smoking and SARS-CoV-2 infection. Furthermore, the same study reported that, among the one hundred seven hospitalized with COVID-19, only 29% were smokers (versus 48% current smokers in the overall population); among the twenty requiring oxygen, only two (10%) were smokers [34]. While smokers should be encouraged to stop smoking, these overall findings suggest a potential protective effect of smoking in SARS-CoV-2 infection and severity. 

A biologically plausible mechanism for the protective effect of smoking observed in COVID-19 infection and mortality could be nicotine’s inhibitory effect on the inflammatory response [26,35]. Through the nicotinic acetylcholine receptor α7 subunit (a7nAChR) on macrophages and B-lymphocytes, the cholinergic nervous system modulates the inflammatory response. As a cholinergic agonist, nicotine inhibits inflammatory cytokines: tumor necrosis factor, interleukin-1, interleukin-6, and high-mobility group box protein 1 (HMGB1). Increased HMGB1 has been associated with more severe COVID-19 clinical outcomes [36]. In other viral disease models, HMGB1 complexes with viral and damage-associated molecules, which are endocytosed by macrophages, and activates inflammasome and cell death pathways [37]. A similar process has been postulated for HMGB1 and SARS-CoV-2 RNA fragments contributing to hyperinflammation in COVID-19 [35]. Like nicotine, the a7nAChR agonist, GTS-21, activates a cholinergic anti-inflammatory response and significantly decreases HMGB1 release, averting the hyperinflammatory state [38]. In mice with hyperoxic lung injury, this anti-inflammatory effect of GTS-21 decreases lung injury [39]. Similarly, in animal models, nicotine protects against acute respiratory distress syndrome induced by lipopolysaccharides. Taken together, nicotine or other cholinergic agonists may favorably affect COVID-19 severity. Nicotine is being evaluated for its efficacy in the prevention of COVID-19 in an ongoing clinical trial [40].

In addition, alcohol use disorder was associated with a decreased relative risk of mortality among infected vs. uninfected patients. Generally, patients with alcohol use disorder are four times more likely to die from pneumonia than nondrinkers [41]. Yet, in COVID-19, alcohol’s effects on the immune system may modulate disease severity. In COVID-19, macrophages release inflammatory cytokines (e.g., HMGB1) that can induce a hyperinflammatory response, which can be fatal [42]. However, alcohol impairs alveolar macrophage release of cytokines and chemokines, as well as phagocytic capacity. Alcohol also decreases neutrophil recruitment and phagocytosis, lymphocyte count, and T cell interferon-gamma and interleukin-12 production. This alcohol-induced diminution in inflammatory cytokines, neutrophil, and lymphocyte response may dampen the hyperinflammatory response and its sequelae, significantly improving mortality in infected patients in comparison to those uninfected. More studies are needed to confirm this novel finding and elucidate its mechanisms.

To further explore the biologic mechanisms associated with lower mortality with smoking and alcohol use disorder, an analysis of the effect of nicotine replacement therapy, and other medications which may affect SARS-CoV-2 infection or severity, is underway. Our analysis of multifactorial interactions affirmed the consistency of the association of current smoking among infected patients with a lower relative risk of mortality.

Our study’s strengths are its national scope, large size, and high-quality laboratory data. By comparing infected and uninfected patients within an integrated healthcare system, we enabled a detailed evaluation of demographic characteristics, comorbidities, time, location, and disease severity to identify risk factors that might influence COVID-19 in an evolving pandemic. To assure study population homogeneity, we reported only the first event of COVID-19 in all patients. Finally, inclusion of the reliable VHA data on smoking [43] and alcohol use disorder [44] provides valuable information and strength to the study.

Our study is limited by its inclusion of exclusively males in the VHA population, a decision based on the low COVID-19 mortality rates and numbers of females in the cohort. We sought to assure adequate numbers to assess the effect of age, race, obesity, and other comorbidities on mortality. With the high rates of comorbidity (and likely polypharmacy) among those infected, it would be helpful to assess the effects and interactions of medication use on mortality. As our study relied solely on SARS-CoV-2 testing to determine COVID-19 status, rather than clinical adjudication, false positive or negative tests could impact the findings. Undiagnosed (untested) seropositivity related to mild or asymptomatic cases poses another source of study bias.

Although we adjusted our analyses for baseline comorbidities and other covariates as a surrogate indicator of utilization, we acknowledge the potential of residual confounding and misclassification bias. We cannot discount that the SARS-CoV-2 test-negative group (screened before hospital admission) represents an enriched population with higher utilization and comparatively poorer outcomes than the test-positive group. Potential differential self-selection for SARS-CoV-2 testing among otherwise healthy smokers also must be carefully considered when interpreting our findings. In this study, alcohol use disorder was defined using ICD-10 codes indicative of alcohol-related health issues coded within two years before the index date. The overall prevalence of alcohol use disorder in the cohort was 18%, which is higher than the previously reported prevalence of alcohol use disorder based on AUDIT-C scores [45] and Fifth Edition of the Diagnostic and Statistical Manual of Mental Disorders (DSM-5) [46] (~11%). The potential influence of self-selection for SARS-CoV-2 testing among alcohol users cannot be excluded. There remains a possibility of “confounding by nonfatal diseases”, which could contribute to the protective effect of smoking. Lastly, as our study population is largely older males, our results may not be fully generalizable to other healthcare systems.

## 5. Conclusions

Comparing SARS-CoV-2-infected versus uninfected men in this large national cohort, we found the key risk factors associated with increased mortality among those infected vs. uninfected to be increased age and overweight/obesity. Current smoking was associated with lower infection rates than observed in never or former smokers. Current smoking or alcohol use disorder were associated with a significantly lower mortality risk in those infected vs. uninfected. As this is an observational study, the findings should be interpreted cautiously. Despite these limitations, the potential protective association of current smoking is supported by a consistent decreased rate of infection and mortality in large observational studies [8,11], a dose-related protective effect of increasing cigarette consumption [32,34], and the biological plausibility of nicotine’s anti-inflammatory effects [26,35]. These findings require further study and elucidation of potential mechanisms. 

## Figures and Tables

**Table 1:** Clinical Characteristics of the study population by SARS-CoV-2 infection status and 30-day mortality.

Characteristic	SARS-CoV-2 Status					
	Infected			Uninfected		
	Dead	Alive	*p* ^¥^	Dead	Alive	*p* ^¥^
	*n* (%)	*n* (%)		*n* (%)	*n* (%)	
	Median (IQR)	Median (IQR)		Median (IQR)	Median (IQR)	
*N* (%)	1520 (7)	21,257 (93)	---	8163 (3)	310,226 (97)	---
Age (year)	76 (16)	60 (24)	<0.0001	74 (14)	66 (19)	<0.0001
≤30	0 (0)	1176 (6)		7 (<1)	9388 (3)	
31–40	2 (<1)	2684 (13)		35 (<1)	27,001 (9)	
41–50	21 (1)	2637 (12)		76 (1)	27,236 (9)	
51–60	69 (5)	4171 (20)		475 (6)	52,105 (17)	
61–70	304 (20)	4852 (23)		1989 (24)	83,164 (27)	
71–80	537 (35)	4261 (20)		3060 (37)	84,926 (27)	
81–90	389 (26)	1162 (5)		1739 (21)	21,590 (7)	
>90	198 (13)	314 (1)		782 (10)	4816 (2)	
Black Race ^^^	532 (35)	7889 (37)	0.10	1588 (19)	73,198 (24)	<0.0001
Latinx ^^^	148 (10)	3709 (17)	<0.0001	662 (8)	31,051 (10)	<0.0001
BMI (kg/m^2^)	27 (8)	30 (8)	<0.0001	25 (8)	29 (8)	<0.0001
<18.5	68 (4)	256 (1)		864 (11)	5281 (2)	
18.5–24.9	467 (31)	3358 (16)		3258 (40)	65,125 (21)	
25–29.9	467 (31)	7115 (33)		2216 (27)	106,208 (34)	
30–34.9	289 (19)	6136 (29)		1081 (13)	79,500 (26)	
35–39.9	144 (9)	2828 (13)		466 (6)	34,797 (11)	
40–44.9	51 (3)	1062 (5)		158 (2)	12,643 (4)	
≥45	34 (2)	502 (2)		120 (1)	6672 (2)	
Alcohol Use Disorder ^^^	152 (10)	3002 (14)	<0.0001	1540 (19)	55,965 (18)	0.056
Smoker ^§^			<0.0001			<0.0001
Never	604 (40)	10,197 (48)		1748 (21)	67,205 (42)	
Former	811 (53)	8548 (40)		4012 (49)	131,564 (42)	
Current	105 (7)	2512 (12)		2404 (29)	111,457 (36)	
Hospitalization^^^	1049 (69)	6100 (29)	<0.0001	4553 (56)	75,240 (24)	<0.0001
LOS (d)	9 (9)	6 (10)	<0.0001	4 (6)	2 (4)	<0.0001
≤7 ^~^	896 (59)	18,654 (88)		6976 (85)	6487 (2)	
>7–14	369 (24)	1191 (6)		828 (10)	5391 (2)	
>14	255 (17)	1412 (7)		359 (4)	5458 (2)	
Mechanical Ventilation ^^^	557 (37)	544 (3)	<0.0001	963 (12)	5458 (2)	<0.0001
Location (USA)			<0.0001			<0.0001
Pacific-Mountain	205 (13)	4053 (19)		1626 (20)	72,970 (24)	
Mid-West	233 (15)	4440 (21)		1787 (22)	62,805 (20)	
East-Coast	1082 (71)	12,764 (60)		4750 (58)	174,451 (56)	
Time (Index, 1 March–10 September)			<0.0001			<0.0001
March	242 (16)	1621 (8)		287 (4)	5,558 (2)	
April	421 (28)	2722 (13)		1182 (14)	19,534 (6)	
May	179 (12)	1669 (8)		1425 (17)	34,482 (11)	
June	195 (13)	3819 (18)		1580 (19)	61,545 (20)	
July	297 (20)	7882 (37)		1702 (21)	91,403 (29)	
August	174 (11)	3361 (16)		1546 (19)	83,980 (27)	
September	12 (1)	183 (1)		441 (5)	13,724 (4)	
Charlson Comorbidity Index			<0.0001			<0.0001
0	374 (25)	11,280 (53)		1036 (13)	138,715 (45)	
1–2	541 (36)	7023 (33)		2171 (27)	107,614 (35)	
3–4	373 (25)	2019 (10)		1979 (24)	41,228 (13)	
5+	230 (15)	935 (4)		2977 (36)	22,669 (7)	
Comorbidity ^						
Asthma	59 (4)	1254 (6)	<0.0001	348 (4)	19,852 (6)	<0.0001
Atherosclerosis	857 (54)	5462 (26)	<0.0001	5233 (64)	109,317 (35)	<0.0001
Cancer	375 (25)	2599 (12)	<0.0001	4017 (49)	62,782 (20)	<0.0001
Chronic Kidney Disease	584 (38)	3208 (15)	<0.0001	3440 (42)	55,028 (18)	<0.0001
Chronic Liver Disease	66 (4)	500 (2)	<0.0001	937 (11)	11,357 (4)	<0.0001
Congestive Heart Failure	425 (28)	2375 (11)	<0.0001	3551 (44)	48,881 (16)	<0.0001
Chronic Obstructive Pulmonary Disease	448 (29)	2986 (14)	<0.0001	3822 (47)	70,300 (23)	<0.0001
Diabetes (Type II)	771 (51)	7098 (33)	<0.0001	3755 (46)	105,024 (34)	<0.0001
Hyperlipidemia	990 (65)	11,617 (55)	<0.0001	5546 (68)	182,297 (59)	<0.0001
Hypertension	1170 (77)	12,160 (57)	<0.0001	6425 (79)	195,340 (63)	<0.0001
Mental Illness	661 (43)	10,031 (47)	0.0052	3936 (48)	158,433 (51)	<0.0001
Sleep Disorder	378 (25)	6053 (28)	0.0025	2037 (25)	90,685 (29)	<0.0001
Substance Abuse	289 (19)	4771 (22)	0.0019	2977 (36)	97,902 (32)	<0.0001

^^^ Referent is the complement group. ^~^ Includes nonhospitalized participants with zero LOS. ^§^ Cigarettes. **^¥^** Chi-square test for independence (categorical), Deuchler–Wilcoxon test (continuous). BMI = body mass index. d = days. SARS-CoV-2 = severe acute respiratory syndrome coronavirus 2. IQR = interquartile range. kg = kilograms. LOS = length of stay. m = meters. USA = United States of America.

## Data Availability

These analyses were performed using raw data that are available only within the US Department of Veterans Affairs firewall in a secure research environment, the VA Informatics and Computing Infrastructure (VINCI). To comply with VA privacy and data security policies and regulatory constraints, only aggregate summary statistics and results of our analyses are permitted to be removed from the data warehouse for publication. The authors have provided detailed results of the analyses in the paper. These restrictions are in place to maintain patient privacy and confidentiality.

## Supplementary Materials

The following are available online at https://www.mdpi.com/article/10.3390/ijerph18168486/s1, Table S1: Selected Characteristics of Male Veterans by SARS-CoV-2 and Mortality Status, Table S2: Adjusted Risk of 30-Day Mortality by SARS-CoV-2 Infection Status and Risk Differences between the SARS-CoV-2 Infection Status. Table S3: Adjusted Risk of 30-Day Mortality by SARS-CoV-2 Infection Status and Risk Difference between the SARS-CoV-2 Infection Status—Stratified Analyses.
