# Supplementary material for: COVID-19-Associated Mortality in US Veterans with and without SARS-CoV-2 Infection
Source: Int J Environ Res Public Health. 2021 Aug 11;18(16):8486. doi: 10.3390/ijerph18168486 (PMC8394601; doi:10.3390/ijerph18168486)
Supplement: Supplementary file 1 [file ijerph-18-08486-s001.zip › ijerph-1297283-SM.pdf]

Table S1. Selected Characteristics of Male Veterans by SARS-CoV-2 and Mortality Status (N = 341,166).

Characteristic	SARS-CoV-2 Status Positive (+) n (%) Median [IQR]	SARS-CoV-2 Status Negative (-) n (%) Median [IQR]	*P*[¥]
Age (y)	62 [24]	66 [19]	<.0001
≤30	1,176 (5)	9,395 (3)	<.0001
31-40	2,686 (12)	27,037 (8)	
41-50	2,658 (12)	27,311 (9)	
51-60	4,240 (19)	52,580 (17)	
61-70	5,156 (23)	85,153 (27)	
71-80	4,798 (21)	87,986 (28)	
81-90	1,551 (7)	23,329 (7)	
>90	512 (2)	5,598 (2)	
Black Race[^]	8,421 (37)	74,786 (23)	<.0001
Latinx[^]	3,857 (17)	31,713 (10)	<.0001
BMI (kg/m^2)	30 [8]	29 [8]	<.0001
<18.5	324 (1)	6,145 (2)	<.0001
18.5-24.9	3,825 (17)	68,384 (21)	
25-29.9	7,582 (33)	108,423 (34)	
30-34.9	6,425 (28)	80,581 (25)	
35-39.9	2,972 (13)	35, 263 (11)	
40-44.9	1,113 (5)	12,801 (4)	
≥45	536 (2)	6,792 (2)	
Alcohol Use Disorder[^]	3,154 (14)	57,505 (18)	<.0001
Smoker[§]			
Never	10,801 (47)	113,860 (36)	<.0001
Former	9,359 (41)	135,576 (43)	
Current	2,617 (11)	68,953 (22)	
Hospitalization[^]	7,149 (31)	79,793 (25)	<.0001

[^]Referent is the complement group. ~Includes non-hospitalized participants with zero LOS. [§]Cigarettes. [¥]Chi-square test for independence (categorical), Deuchler-Wilcoxon test (continuous). BMI = Body mass index. d = Days. SARS-CoV-2= severe acute respiratory syndrome coronavirus. IQR = Interquartile range. kg = kilograms. LOS = Length of stay. m = Meters. USA = United States of America. y = Years.

Table S1. (cont.) Selected Characteristics of Male Veterans by SARS-CoV-2 and Mortality Status.

Characteristic	SARS-CoV-2 Status		
	**Positive (+) n (%) Median [IQR]**	**Negative (-) n (%) Median [IQR]**	$P^{¥}$
LOS (d)			
≤7~	19,550 (86)	305,324 (96)	<.0001
>7-14	1,560 (7)	7,315 (2)	
>14	1,667 (7)	5,750 (2)	
Mechanical Ventilation^	1,101 (5)	6,421 (2)	<.0001
Location (USA)			
Pacific-Mountain	4,258 (19)	74,596 (23)	<.0001
Mid-West	4,673 (21)	64,593 (20)	
East-Coast	13,846 (61)	179,200 (56)	
Time (Index, 3/1 – 9/10)			
March	1,863 (8)	5,845 (2)	<.0001
April	3,143 (14)	20,716 (7)	
May	1,848 (8)	35,907 (11)	
June	4,014 (18)	63,125 (20)	
July	8,179 (36)	93,106 (29)	
August	3,535 (16)	85,525 (27)	
September	195 (1)	14,165 (4)	
Charlson Comorbidity Index			
0	11,656 (51)	139,751 (44)	<.0001
1-2	7,564 (33)	109,785 (34)	
3-4	2,392 (11)	43,207 (14)	
5+	1,165 (5)	25,646 (8)	

^Referent is the complement group. ~Includes non-hospitalized participants with zero LOS. §Cigarettes. ¥Chi-square test for independence (categorical), Deuchler-Wilcoxon test (continuous). BMI = Body mass index. d = Days. SARS-CoV-2= severe acute respiratory syndrome coronavirus. IQR = Interquartile range. kg = kilograms. LOS = Length of stay. m = Meters. USA = United States of America. y = Years.

Table S1. (cont.) Selected Characteristics of Male Veterans by SARS-CoV-2 and Mortality Status.

Characteristic	COVID-19 Status		
	Positive (+) n (%) Median [IQR]	Negative (-) n (%) Median [IQR]	$P^{¥}$
Comorbidity^			
Asthma	1,313 (6)	20,200 (6)	0.0005
Atherosclerosis	6,289 (28)	114,550 (36)	<.0001
Cancer	2,974 (13)	66,799 (21)	<.0001
Chronic Kidney Disease	3,792 (17)	58,468 (18)	<.0001
Chronic Liver Disease	566 (2)	12,294 (4)	<.0001
Congestive Heart Failure	2,800 (12)	52,432 (16)	<.0001
Chronic Obstructive Pulmonary Disease	3,434 (15)	74,122 (23)	<.0001
Diabetes (Type II)	7,869 (35)	108,779 (34)	0.24
Hyperlipidemia	12,607 (55)	187,842 (59)	<.0001
Hypertension	13,330 (59)	201,765 (63)	<.0001
Mental Illness	10,692 (47)	162,369 (51)	<.0001
Sleep Disorder	6,431 (28)	92,721 (29.)	0.0044
Substance Abuse	5,060 (22)	100,879 (32)	<.0001

^Referent is the complement group. ~Includes non-hospitalized participants with zero LOS. §Cigarettes. ¥Chi-square test for independence (categorical), Deuchler-Wilcoxon test (continuous). BMI = Body mass index. d = Days. SARS-CoV-2= severe acute respiratory syndrome coronavirus. IQR = Interquartile range. kg = kilograms. LOS = Length of stay. m = Meters. USA = United States of America. y = Years

Table S2. Adjusted Risk of 30-Day Mortality by SARS-CoV-2 Infection Status and Risk Difference between the SARS-CoV-2 Infection Status.

Characteristic	SARS-CoV-2 Status						Effect (+ | −)	
	Infected			Uninfected				
	Dead n (%)	Alive n (%)	aRR (95%CI) †	Dead n (%)	Alive n (%)	aRR (95%CI)†	Δ_{aRR}♦ %	P_{Int}†¥
Age (years)								
≤60	92 (6)	10,668 (50)	1.0 Referent	593 (7)	115,730 (37)	1.0 Referent	---	---
61-70	304 (20)	4,852 (23)	3.4 (2.7-4.2)	1,989 (24)	83,164 (27)	1.9 (1.8-2.1)	+73	<.0001
71-80	537 (35)	4,261 (20)	6.1 (4.9-7.5)	3,060 (37)	84,926 (27)	2.6 (2.4-2.9)	+131	<.0001
>80	587 (39)	1,476 (7)	13 (11-16)	2,521 (31)	26,406 (9)	5.8 (5.3-6.3)	+132	<.0001
P_{Trend}*	---	---	<.0001	---	---	<.0001	---	---
Black Race^	532 (35)	7,889 (37)	.90 (.81-.9997)	1,588 (19)	73,198 (24)	.89 (.83-.93)	+3	.66
Latinx^	148 (10)	3,709 (17)	.98 (.84-1.2)	662 (8)	31,051 (10)	1.1 (.993-1.2)	-9	.33
BMI (kg/m²)								
18.5-24.9	467 (31)	3,358 (16)	1.0 Referent	3,258 (40)	65,126 (21)	1.0 Referent	---	---
<18.5	68 (4)	256 (1)	1.2 (.995-1.6)	864 (11)	5,281 (2)	2.2 (2.1-2.4)	-76	<.0001
25-29.9	467 (31)	7,115 (33)	.87 (.77-.97)	2,216 (27)	106,207 (34)	.57 (.55-.60)	+51	<.0001
30-34.9	289 (19)	6,136 (29)	.90 (.77-1.04)	1,081 (13)	79,500 (26)	.44 (.41-.47)	+103	<.0001
35-39.9	144 (9)	2,828 (13)	1.1 (.89-1.3)	466 (6)	34,797 (11)	.42 (.38-.46)	+160	<.0001
40-44.9	51 (3)	1,062 (5)	1.1 (.83-1.6)	158 (2)	12,643 (4)	.38 (.32-.44)	+203	<.0001
≥45	34 (2)	502 (2)	2.0 (1.4-2.8)	120 (1)	6,672 (2)	.51 (.43-.61)	+290	<.0001
Alcohol Use Disorder^	152 (10)	3,002 (14)	.91 (.77-1.1)	1,540 (19)	55,965 (18)	1.4 (1.3-1.45)	-51	<.0001
Smoker§								
Never	604 (40)	10,197 (48)	1.0 Referent	2,403 (29)	111,457 (36)	1.0 Referent	---	---
Former	811 (53)	8,548 (40)	1.0 (.91-1.1)	4,012 (49)	131,564 (42)	.98 (.94-1.03)	+2	.76
Current	105 (7)	2,512 (12)	.81 (.66-.989)	1,748 (21)	67,205 (22)	1.3 (1.2-1.4)	-56	<.0001

*Likelihood ratio test for linear trend. ^Referent is the complement group. §Cigarettes. †Adjusted for Age (≤60, 61-70, 71-80, >80), Location (Pacific-Mountain, Mid-West, East-Coast), Time (March, April, May, June, July, August/September), and Charlson Comorbidity Index (1, 1-2, 3-4, 5+), unless the indicated characteristic. ♦Percentage change in RR for SARS-COV-2 (+) vs. (-) on logarithmic scale. ¥Unrestricted test for interaction on the multiplicative scale. aRR = Adjusted relative risk (Le Cam-Stein shrinkage). BMI = Body mass index (kg/m²). CCI = Charlson Comorbidity Index
CI = Confidence interval. SARS-CoV-2= severe acute respiratory syndrome coronavirus 2. kg = kilograms. m = Meters.

Table S2. (cont.) Adjusted Risk of 30-Day Mortality by SARS-CoV-2 Infection Status and Risk Difference between the SARS-CoV-2 Infection Status.

Characteristic	SARS-CoV-2 Status						Effect (+ | −)	
	Infected			Uninfected				
	Dead n (%)	Alive n (%)	aRR (95%CI)†	Dead n (%)	Alive n (%)	aRR (95%CI)†	Δ_{aRR}♦ %	P_{Int}†¥
Location (USA)								
Pacific-Mountain	205 (13)	4,053 (19)	1.0 Referent	1,626 (20)	72,970 (24)	1.0 Referent	---	---
Mid-West	233 (15)	4,440 (21)	.90 (.74-1.1)	1,787 (22)	62,806 (20)	1.1 (1.03-1.2)	-23	.038
East-Coast	1,082 (71)	12,764 (60)	1.2 (1.0002-1.3)	4,750 (58)	174,450 (56)	1.1 (1.02-1.1)	+8	.35
Time (Index)								
March	242 (16)	1,621 (8)	1.0 Referent	287 (4)	5,558 (2)	1.0 Referent	---	---
April	421 (28)	2,722 (13)	.81 (.70-.93)	1,182 (14)	19,534 (6)	.85 (.75-.95)	-5	.60
May	179 (12)	1,669 (8)	.63 (.52-.75)	1.425 (17)	34,482 (11)	.57 (.50-.63)	+11	.36
June	195 (13)	3,819 (18)	.49 (.41-.59)	1,580 (19)	61,545 (20)	.39 (.34-.43)	+27	.031
July	297 (20)	7,882 (37)	.37 (.32-.44)	1,702 (21)	91,404 (29)	.30 (.27-.34)	+24	.032
Aug/Sep	186 (12)	3,544 (17)	.41 (.35-.49)	1,987 (24)	97,703 (31)	.27 (.24-.30)	+54	<.0001
CCI								
0	376 (25)	11,280 (53)	1.0 Referent	1,036 (13)	138,715 (45)	1.0 Referent	---	---
1-2	541 (36)	7,023 (33)	1.2 (1.1-1.4)	2,171 (27)	107,614 (35)	1.7 (1.6-1.8)	-38	<.0001
3-4	373 (25)	2,019 (10)	1.7 (1.5-1.9)	1,979 (24)	41,228 (13)	3.0 (2.8-3.2)	-78	<.0001
5+	230 (15)	935 (4)	2.0 (1.8-2.3)	2,977 (36)	22,669 (7)	7.3 (6.8-7.8)	-262	<.0001
P_{Trend}*	---	---	<.0001	---	---	<.0001	---	---

*Likelihood ratio test for linear trend. ^Referent is the complement group. §Cigarettes. †Adjusted for Age (≤60, 61-70, 71-80, >80), Location (Pacific-Mountain, Mid-West, East-Coast), Time (March, April, May, June, July, August/September), and Charlson Comorbidity Index (0, 1-2, 3-4, 5^+), unless the indicated characteristic. ♦Percentage change in RR for SARS-CoV-2 (+) vs. (-) on logarithmic scale. ¥Unrestricted test for interaction on the multiplicative scale. aRR = Adjusted relative risk (Le Cam-Stein shrinkage). BMI = Body mass index (kg/m^2). CCI = Charlson Comorbidity Index
CI = Confidence interval. SARS-CoV-2= severe acute respiratory syndrome coronavirus 2. kg = kilograms. m = Meters.

Table S3. Adjusted Risk of 30-Day Mortality by SARS-CoV-2 Infection Status and Risk Difference between the SARS-CoV-2 Infection Status – Stratified Analyses.

Stratum	SARS-CoV-2 Status						Effect (+ | −)			↕ Stratum	
	Infected			Uninfected			Δ_{aRR}[♦] %	P_{Int}[†¥]		$P_{\Delta V}$[†⅄]	
	Dead[^] n (%)	Alive[^] n (%)	aRR (95%CI)[†]	Dead[^] n (%)	Alive[^] n (%)	aRR (95%CI)[†]		Multiplicity[‡]			
	Age (> 70 vs. ≤ 70 y)						---	$\overline{Adj}$	*Adj*	$\overline{Adj}$	*Adj*
Smoker +[§]	59 (56)	431 (17)	3.2 (2.2-4.7)	832 (48)	14,408 (21)	1.8 (1.6-1.9)	+83	.0019	.0019	.60	.60
Smoker −[§]	1,065 (75)	5,306 (28)	4.0 (3.6-4.5)	4,749 (74)	96,924 (40)	2.4 (2.3-2.6)	+64	<.0001	<.0001		
Black	334 (63)	1,639 (21)	3.4 (2.9-4.0)	922 (58)	18,253 (25)	2.2 (2.0-2.4)	+53	<.0001	<.0001	.026	.10
White	790 (80)	4,098 (31)	4.4 (3.8-5.1)	4,659 (71)	93,079 (39)	2.2 (2.0-2.3)	+104	<.0001	<.0001		
BMI ≥ 25	663 (67)	4,044 (23)	3.6 (3.2-4.0)	2,616 (65)	79,473 (33)	2.0 (1.9-2.2)	+76	<.0001	<.0001	.14	.42
BMI < 25	461 (86)	1,693 (47)	4.3 (3.5-5.5)	2,965 (72)	31,859 (45)	2.0 (1.9-2.1)	+117	<.0001	<.0001		
ETOH +	86 (57)	515 (17)	3.1 (2.3-4.3)	629 (41)	10,957 (20)	1.5 (1.4-1.7)	+107	<.0001	<.0001	.15	.30
ETOH −	1,038 (76)	5,222 (29)	4.1 (3.7-4.6)	4,952 (75)	100,375 (39)	2.6 (2.4-2.7)	+60	<.0001	<.0001		
	Black vs. White						---	$\overline{Adj}$	*Adj*	$\overline{Adj}$	*Adj*
Age > 70 y	334 (30)	1,639 (29)	.88 (.78-.988)	922 (17)	18,253 (16)	.93 (.87-.995)	-6	.40	1.0	.44	1.0
Age ≤ 70 y	198 (50)	6,250 (40)	.97 (.79-1.2)	666 (26)	54,945 (28)	.80 (.74-.88)	+20	.10	.73		
Smoker +	40 (38)	983 (39)	.92 (.60-1.4)	373 (21)	17,676 (26)	.83 (.74-.93)	+11	.65	1.0	1.0	1.0
Smoker −	492 (35)	6,906 (37)	.90 (.80-.997)	1,215 (19)	55,522 (23)	.89 (.84-.95)	+<1	1.0	1.0		
BMI ≥ 25	343 (35)	6,431 (36)	.94 (.82-1.07	645 (16)	54,194 (23)	.76 (.70-.83)	+24	.0087	.070	.18	.71
BMI < 25	189 (35)	1,468 (41)	.83 (.70-.98)	943 (23)	19,004 (27)	.86 (.80-.92)	-4	.67	1.0		
ETOH +	62 (41)	1,253 (42)	.78 (.55-1.1)	365 (24)	16,465 (29)	.74 (.66-.83)	+5	.79	1.0	1.0	1.0
ETOH −	470 (34)	6,636 (36)	.91 (.82-1.02)	1,223 (18)	56,733 (22)	..91 (.85-.96)	+1	.88	1.0		
	BMI (≥ 25 vs. <25)						---	$\overline{Adj}$	*Adj*	$\overline{Adj}$	*Adj*
Age > 70 y	663 (59)	4,044 (70)	.85 (.76-.95)	2,616 (47)	19,473 (71)	.47 (.45-.49)	+82	<.0001	<.0001	.056	.22
Age ≤ 70 y	322 (81)	13,599 (88)	1.0 (.80-1.3)	1,425 (55)	160,346 (81)	.42 (.39-.45)	+142	<.0001	<.0001		
Black	343 (64)	6,421 (82)	.92 (.77-1.1)	645 (41)	54,194 (74)	.40 (.36-.44)	+132	<.0001	<.0001	.071	.21
White	642 (65)	11,222 (84)	.85 (.75-.95)	3,396 (52)	185,625 (78)	.46 (.44-.48)	+84	<.0001	<.0001		
Smoker +	61 (58)	1,860 (74)	.94 (.59-1.5)	722 (41)	45,162 (67)	.49 (.45-.54)	+90	.0072	.014	.98	.98
Smoker −	924 (65)	15,983 (84)	.87 (.78-.96)	3,319 (52)	194,657 (80)	.45 (.43-.47)	+92	<.0001	<.0001		
ETOH +	91 (60)	2,366 (79)	.83 (.60-1.1)	741 (48)	38,923 (70)	.55 (.50-.60)	+51	.014	.014	.15	.31
ETOH −	894 (65)	15,277 (84)	.88 (.79-.98)	3,300 (50)	200,897 (79)	.44 (.42-.47)	+98	<.0001	<.0001		

[^]Non-referent group of the indicated comparison factor. [†]Adjusted for Age (≤60, 61-70, 71-80, >80), Location (Pacific-Mountain, Mid-West, East-Coast), Time (March, April, May, June, July, August/September), and Charlson Comorbidity Index (0, 1-2, 3-4, 5^{+}). [♦]Percentage change in RR for SARS-CoV-2 (+) vs. (-) on logarithmic scale. [¥]Unrestricted test for interaction on the multiplicative scale for SARS-CoV-2 status comparison. [‡]Unadjusted ($\overline{Adj}$) | Adjusted (*Adj*) for multiplicity within the indicated comparison factor using the Hochberg step-up procedure. [⅄]P-value for absolute difference (vertical stratum effect). [§]Cigarettes. aRR = Adjusted relative risk (Le Cam-Stein shrinkage). BMI = Body mass index (kg/m^2, kg = kilograms. m = Meters). CI = Confidence interval. SARS-CoV-2= severe acute respiratory syndrome coronavirus 2. y = Years.

Table S3. (cont.) Adjusted Risk of 30-Day Mortality by SARS-CoV-2 Infection Status and Risk Difference between the SARS-CoV-2 Infection Status – Stratified Analyses.

Stratum	SARS-CoV-2 Status: Infected Dead^ n (%)	Infected Alive^ n (%)	Infected aRR (95%CI)†	Uninfected Dead^ n (%)	Uninfected Alive^ n (%)	Uninfected aRR (95%CI)†	Effect (+ | −): Δ_{aRR}♦ %	Effect (+ | −): P_{Int}†¥ Multiplicity‡ $\overline{Adj}$	Effect (+ | −): P_{Int}†¥ Multiplicity‡ *Adj*	↕ Stratum: $P_{\Delta V}$†⅄ Multiplicity‡ $\overline{Adj}$	↕ Stratum: $P_{\Delta V}$†⅄ Multiplicity‡ *Adj*
Current Smoker (Yes vs. No)§							---	$\overline{Adj}$	*Adj*	$\overline{Adj}$	*Adj*
Age > 70 y	59 (5)	431 (8)	.86 (.67-1.1)	832 (15)	14,408 (13)	1.3 (1.2-1.4)	-47	.0027	.011	.65	1.0
Age ≤ 70 y	46 (12)	2,081 (13)	.75 (.55-1.1)	916 (35)	52,797 (27)	1.3 (1.2-1.4)	-77	.0004	.0031		
Black	40 (8)	983 (12)	.79 (.58-1.1)	373 (23)	17,676 (24)	1.2 (1.1-1.4)	-56	.0099	.030	1.0	1.0
White	65 (7)	1,529 (11)	.84 (.66-1.1)	1,375 (21)	49,529 (21)	1.3 (1.2-1.4)	-55	.0006	.0036		
BMI ≥ 25	61 (6)	1,860 (11)	.81 (.63-1.04)	722 (18)	45,162 (19)	1.2 (1.1-1.3)	-52	.0022	.0011	.75	1.0
BMI < 25	44 (8)	652 (18)	.73 (.54-.994)	1,026 (25)	22,043 (31)	1.0 (.93-1.1)	-36	.054	.11		
ETOH +	32 (21)	762 (25)	.97 (.65-1.4)	636 (41)	23,205 (41)	1.2 (1.1-1.3)	-20	.39	.39	.28	1.0
ETOH −	73 (5)	1,750 (10)	.80 (.64-1.0)	1,112 (17)	44,000 (17)	1.3 (1.2-1.3)	-56	.0002	.0015		
Alcohol Use Disorder (Yes vs. No)							---	$\overline{Adj}$	*Adj*	$\overline{Adj}$	*Adj*
Age > 70 y	86 (8)	515 (9)	.92 (.75-1.1)	629 (11)	10,957 (9)	1.2 (1.1-1.3)	-30	.017	.051	.11	.32
Age ≤ 70 y	66 (17)	2,487 (16)	.88 (.69-1.2)	911 (35)	45,008 (23)	1.6 (1.4-1.7)	-75	<.0001	.0004		
Black	62 (12)	1,253 (16)	.85 (.66-1.1)	365 (23)	16,465 (22)	1.3 (1.1-1.4)	-48	.0079	.031	.97	.97
White	90 (9)	1,749 (13)	.95 (.78-1.2)	1,175 (18)	39,500 (17)	1.4 (1.3-1.5)	-49	.0003	.0015		
BMI ≥ 25	91 (9)	2,366 (13)	.85 (.69-1.04)	741 (18)	38,923 (16)	1.5 (1.3-1.6)	-72	<.0001	<.0001	.0054	.022
BMI < 25	61 (11)	636 (18)	.99 (.76-1.3)	799 (19)	17,042 (24)	1.0 (.95-1.1)	-4	.77	.77		
Smoker +	32 (30)	762 (30)	1.1 (.72-1.8)	636 (36)	23,205 (35)	1.3 (1.2-1.4)	-14	.59	1.0	.35	.70
Smoker −	120 (8)	2,240 (12)	.91 (.76-1.1)	904 (14)	32,760 (13)	1.3 (1.2-1.4)	-45	.0001	.0008		

^Non-referent group of the indicated comparison factor. †Adjusted for Age (≤60, 61-70, 71-80, >80), Location (Pacific-Mountain, Mid-West, East-Coast), Time (March, April, May, June, July, August/September), and Charlson Comorbidity Index (0, 1-2, 3-4, 5+). ♦Percentage change in RR for SARS-COV-2 (+) vs. (-) on logarithmic scale. ¥Unrestricted test for interaction on the multiplicative scale for SARS-CoV-2 status comparison. ‡Unadjusted ($\overline{Adj}$) | Adjusted (*Adj*) for multiplicity within the indicated comparison factor using the Hochberg step-up procedure. ⅄P-value for absolute difference (vertical stratum effect). §Cigarettes. aRR = Adjusted relative risk (Le Cam-Stein shrinkage). BMI = Body mass index (kg/m^2, kg = kilograms. m = Meters). CI = Confidence interval. SARS-CoV-2= severe acute respiratory syndrome coronavirus 2. y = Years.
